# Preparation of Activated Carbon Supported Bead String Structure Nano Zero Valent Iron in a Polyethylene Glycol-Aqueous Solution and Its Efficient Treatment of Cr(VI) Wastewater

**DOI:** 10.3390/molecules25010047

**Published:** 2019-12-21

**Authors:** Chunlei Jiao, Xiao Tan, Aijun Lin, Wenjie Yang

**Affiliations:** 1 Department of Environmental Science and Engineering, Beijing University of Chemical Technology, Beijing 100029, China; jiaocl@ihep.ac.cn (C.J.); tanxiaobuct@163.com (X.T.);; 2 Institute of High Energy Physics, Chinese Academy of Sciences, Beijing 100049, China; 3 College of Renewable Energy, North China Electric Power University, Beijing 102206, China; 4 Chinese Academy for Environmental Planning, Beijing 100012, China

**Keywords:** bead string structure, nZVI, polyethylene glycol, Cr(VI) wastewater

## Abstract

Nanometer zero-valent iron (nZVI) has been widely used in the treatment of heavy metals such as hexavalent chromium (Cr(VI)). A novel composite of bead string-structured nZVI on modified activated carbon (nZVI–MAC) is prepared here, using polyethylene glycol as the stable dispersant rather than traditional ethanol during the loading process. The microstructure characterization shows that nZVI particles are loaded on MAC with a bead string structure in large quantity and stably due to the addition of hydroxyl functional groups on the surface by polyethylene glycol. Experiments on the treatment of Cr(VI) in wastewater show that the reaction process requires only 20 min to achieve equilibrium. The removal rate of Cr(VI) with a low concentration (80–100 mg/L) is over 99% and the maximum saturation removal capacity is up to 66 mg/g. The system converts Cr(VI) to trivalent chromium (Cr(III)) through an oxidation-reduction effect and forms an insoluble material with iron ions by coprecipitation, which is then adsorbed on the surface of the nZVI–MAC. The process conforms to the quasi-second order adsorption kinetics equation (mainly chemical adsorption process).

## 1. Introduction

Chromium is widely used in lots of industrial productions such as paper making, electroplating, dye manufacturing, leather tanning and paint manufacturing [1,2,3]. Due to loopholes in mining, smelting, use and disposal processes, chromium has caused many incidences of water contamination in industrialized areas, especially in China’s Bohai Rim region with developed industry [3,4]. Chromium primarily exists as Cr(III) and Cr(VI) in the natural environment, and Cr(VI) is more soluble and toxic than Cr(III) [5,6]. Cr(VI) can cause a series of health problems ranging from skin irritation to lung carcinomas [3,7]. The US Environmental Protection Agency (EPA) issues the national primary drinking water regulations, which stipulate a maximum concentration of 0.1 mg/L total chromium in drinking water [8]. The treatment of Cr(VI) wastewater, therefore, has become an exigent environmental issue.

nZVI has recently become a suitable alternative for the treatment of Cr(VI)-contaminated water due to its high reduction capacity and large specific surface area, indicating a high efficiency and low environmental cost [9,10,11]. However, limitations remain for applying nZVI technology to contaminant control due to the agglomeration tendency of nZVI particles [12,13]. The nZVI particles are prone to agglomeration, causing obvious decreases in the reduction capacity and specific surface area of the particles [3,12,14,15]. Moreover, the reaction of nZVI and Cr(VI) may be inhibited by their precipitated product, which is deposited on the metal surface and thus limits further reactions. Hydrogen ions (H^+^) -have a significant effect on Cr(VI) removal, requiring the solution to be adjusted to an acidic level (pH ≤6) in advance to ensure a high efficiency of nZVI [16,17]. This fact limits nZVI application and generates higher application costs. To address these issues, researchers have attempted to use different types of materials such as grapheme [18,19], biochar [20], chitosan [21], mesoporous silica [22], bentonite [23], kaolinite [24], sepiolite [25], olive stones [26] and activated carbon [4,16,27] to stabilize nZVI as a support by decreasing particle agglomeration and, therefore, improving its application scope.

Many efforts in previous studies have focused on developing new activated carbon carriers [16,28,29]. Due to its low-cost, large surface area and micropores, activated carbon is considered a good carrier of nanomaterials. Many studies have reported the loading of nZVI on activated carbon to maximize the effect of nZVI [8,16,30]. Wu et al. [16] successfully loaded nZVI on activated carbon and demonstrated that a micro-electrolysis effect between iron and carbon plays a vital role in removing Cr(VI). The reaction rate is 10 times that of a traditional adsorption reaction, and the removal rate of chromium can reach 99.5%. Qiu et al. used cellulose for preparation of new activated carbon particles with iron and explored the effects of different carbonization temperatures on material properties, and found that the magnetic carbon synthesized at 700 °C carbonization temperature had a greater chromium(VI) removal capacity (22.8 mg/g) [8]. Zhu et al. prepared magnetic carbon nanocomposite fabrics by microwave-assisted heating; these fabrics served as advanced adsorbents for the removal of Cr(VI) with a much higher removal capacity of 3.74 mg/g compared to 0.32 mg/g for cotton fabrics and 0.46 mg/g for carbon fabrics [30]. Tang et al. successfully prepared a magnetic iron nanoparticle-doped ordered mesoporous carbon material for highly effective Cr(VI) removal with a high surface area of 679.4 m^2^/g and 4.42 wt% magnetic nanoparticles [31]. Additionally, in view of the disadvantage of easy agglomeration of nZVI, recent studies [32] have added polyethylene glycol as a surfactant in the preparation process to increase the stability of nZVI and obtained a good effect. We tried in the past to load nZVI on coconut shell biochar and found that not only the dispersion of nZVI was improved, but also the treatment effect of the material on hexavalent chromium was significantly improved by adding polyethylene glycol [33].

Based on the high removal rate of Cr(VI) by nZVI with carbon materials, this study aims to design an improved preparation method of C–Fe^0^, in which polyethylene glycol completely replaces ethanol as a solvent and dispersant during the loading process. The specific objectives are: (1) modification of activated carbon by hydrothermal synthesis; (2) preparation of nZVI–MAC by liquid phase reduction with polyethylene glycol–H_2_O as a reaction solution; (3) evaluation of key factors affecting the treatment of Cr(VI); (4) discussion of possible mechanisms of removing Cr(VI) by nZVI–MAC. To our knowledge, few studies have reported the complete substitution of polyethylene glycol for ethanol as a reaction solution for the synthesis of nZVI.

## 2. Results and Discussion

### 2.1. Characterization of nZVI–MAC

**Figure 1:**
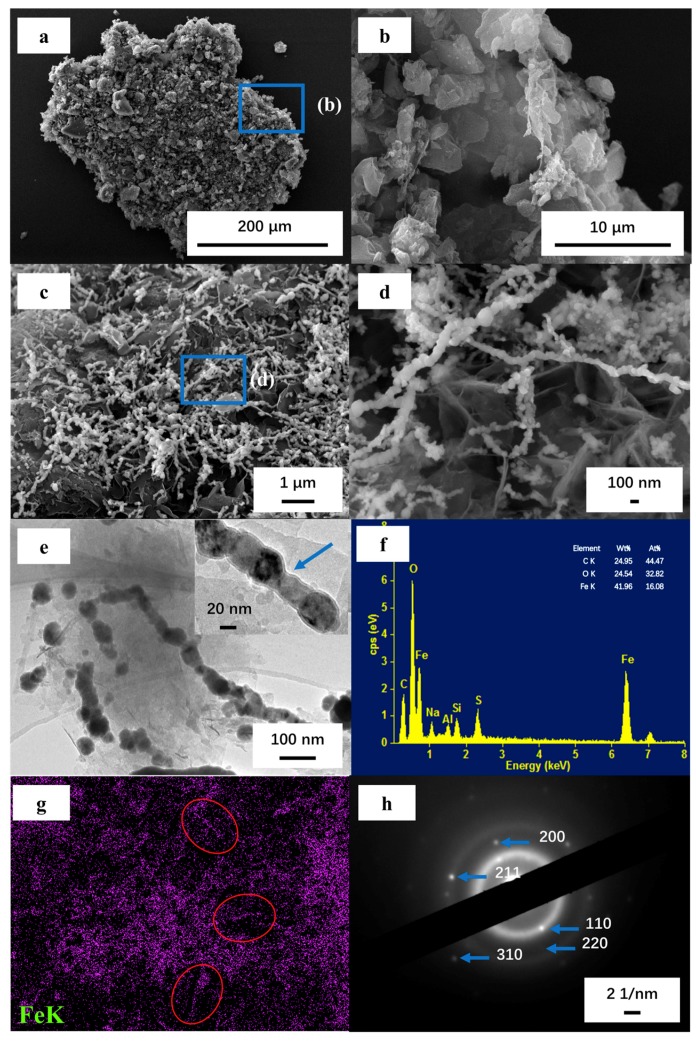
(**a**,**b**) Scanning electron microscope (SEM) images of MAC (**b** is the amplification image of the corresponding dotted box in **a**); (**c**,**d**) SEM images of nZVI–MAC (**d** is the amplification image of the corresponding dotted box in **c**); (**e**) HRTEM image of nZVI–MAC; (**f**) Energy dispersive spectrum(EDS) spectrum of nZVI–MAC; (**g**) EDS mapping of (**c**); and (**h**) SAED image of nZVI–MAC.

The following steps were involved in the synthesis of nZVI–MAC (see ESI, Figure S1). MAC was prepared from activated carbon using a modified hydrothermal synthesis method [34], and then carbon particles were self-generated on the surface of activated carbon via a hydrothermal reaction with microcrystalline cellulose as the carbon source and citric acid as the catalyst (as described in Figure 1a,b). Liu et al. [35] believed that microcrystalline cellulose played a role in providing nucleophile group functions here, which enabled the formation of carbon microspheres and the appendage of carbon particles with activated carbon. Subsequently, the reduction of ferrous ions occurred in the pores and on the surface of MAC in the presence of a reductant (NaBH_4_) in the polyethylene glycol–H_2_O solution system, and then, spherical nZVI particles were formed. Previous studies had nZVI with similar structure also appear, which Wu et al. considered to be caused by a magnetic interaction of nZVI, but they used a traditional ethanol-aqueous solution system in material synthesis [16,36].

Shown in Figure 1c,d, a large number of nZVI chains were twined on the surface of MAC. The presence of carbon particles caused the traditional activated carbon to have a greater specific surface area and more attachment sites, resulting in more nZVI chains being tightly attached to the activated carbon surface, which endows the material with high reducing activity and adsorption capacity. Comparing the specific surface area between the traditional activated carbon and the modified activated carbon, it also can be found that the specific surface area and pore volume of the material can be improved by the modification process (see ESI, Table S1). The high-resolution transmission electron microscopy (HRTEM) images (Figure 1e) of the material show that the chain structure formed by nZVI on MAC is a bead string structure, which is a subtle difference from previous studies [36]. Additionally, a large number of spherical nZVI particles remain that did not assemble into chains, which is not clearly illustrated on the surface of the material. Further HRTEM observations indicated that nZVI had a thin layer of iron oxide on its surface [37] (the blue arrow in Figure 1e), indicating its core-shell structure.

During our past studies, it was found that polyethylene glycol did have a great improvement in enhancing the stability of nZVI, but excessive polyethylene glycol actually affected the exposure of nZVI to pollutants, which weakened the effect of the material [33]. ZVI is still used as an electron source in the treatment of Cr(VI), while the oxide shell only promotes the adsorption of Cr(VI) [38]. This microstructure is similar to that of ZVI particles [39,40,41] prepared by chemical agents. Illustrated in Figure 1g, the observed microscopic area was evenly distributed on the surface of iron, which indicated that the load of nZVI was very uniform without large area agglomeration. Moreover, this figure clearly shows that the iron nanoparticles are arranged in a chain (shown in the red circles) and that none of the particles are in contact with each other, which corresponds to the bead string structure in Figure 1e. The iron nanoparticles may be connected via the carbon and oxygen elements. Additionally, the iron content reached 41.96% in the material, thus endowing the material with a strong activity. According to the design of the material preparation process, the theoretical content of iron is 60%, demonstrating that some of the iron was lost during the washing process. Moreover, the particle size of the composite material is about 18.84 μm, which is based on the carrier particle size of MAC (see ESI, Figure S6).

To determine the nanoparticle morphology in MAC and nZVI–MAC as well as the corresponding crystalline structure, selected area electron diffraction (SAED) and X-ray diffraction (XRD) characterizations were conducted. Figure 1h shows the nanoparticle structure of nZVI–MAC. Nanoparticles on the material surface are polycrystalline and have a body-centred cubic structure, including the Fe crystal planes of (110, 200, 211_1_, 220 and 310) (PDF#06-0696). Added to the ZVI crystal planes corresponding to the SAED patterns, Figure S2b also shows the crystal planes of iron oxide, including (211_2_, 222) (PDF#85-0987), Further, it was suggested that the thin layer of nZVI (Figure 1e) was iron oxide or iron hydroxide, possibly due to the use of polyethylene glycol or oxidation during the preparation of nZVI after short exposure to air. Giorgio et al. studied the oxidation mechanism of nZVI and adopted the shrinking-core mechanism, believing that during the reaction with oxygen and water, the core of nZVI would undergo uniform contraction and generate the structure of the oxidation shell; during the reaction, the structure of the individual particles did not change [42].

Weak peaks (101, 111) (PDF#85-0871) of Fe3C appear in the XRD spectra of the composite material, confirming that a small amount of iron and carbon is crystallized during the loading process.

The crystalline structure of MAC before unloading the iron is shown in Figure S2a. The characteristic peaks (002, 100, 102, 103) of the graphite (PDF#65-6212) appear in the XRD spectrum of MAC compared to the unmodified activated carbon material. Interestingly, the spectrum also reveals a structure similar to fullerite (C60 or C70), such as (201, 401) (PDF#49-1721) and (113, 211, 009) (PDF#50-1363). It is possible that the carbons in cellulose spontaneously bond with each other during the reduction process of the hydrothermal reaction, forming a spherical structure similar to that of fullerite, rather than undergoing a nucleophilic reaction with carbon in the activated carbon. Compared with MAC, the nZVI–MAC crystal structure after being loaded with iron changed greatly, and four characteristic peaks of graphite showed a greater degree of weakening, with some characteristic peaks (such as 100) disappearing.

**Figure 2:**
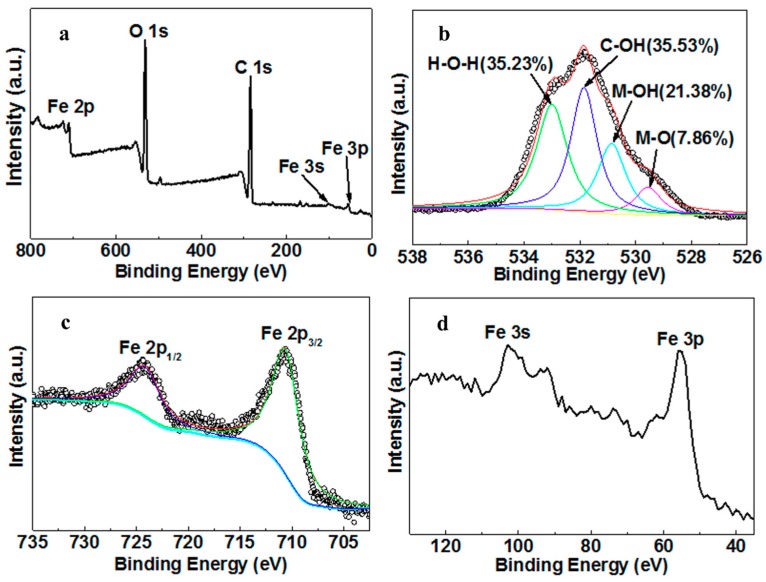
XPS spectra of the nZVI–MAC: (**a**) full spectrum; (**b**) O 1s; (**c**) Fe 2p; (**d**) Fe 3s and Fe 3p.

The X-ray photoelectron spectroscopy (XPS) wide scan profiles show photoelectron lines at binding energies of approximately 56, 103, 285.8, 532, and 711 eV, which are attributed to Fe 3p, Fe 3s, C 1s, O 1s, and Fe 2p, respectively (Figure 2a). Shown in Figure 2b, the O 1s spectra were divided into several typical oxygen-containing functional peaks (529.55, 530.85, 531.85, 533 eV), which might represent the iron oxide bond (M–O), the metal-hydroxyl bond (M–OH) in iron hydroxide, the hydroxyl in nZVI–MAC (C–OH), and unremoved free water (H–O–H), respectively [35,43,44]. The presence of so many hydroxyl groups on the surface of the material is due to the addition of polyethylene glycol during preparation [33]. Supplementary to providing a solvent with strong dispersing ability rather than traditional ethanol, polyethylene glycol also acts as a surfactant to improve the surface polarity of materials and load more nZVI particles without agglomeration. Seen in the spectra of Fe 2p (Figure 2c), the twin peaks of the 710.85 eV (Fe 2p_3/2_) and 724.5 eV (Fe 2p_1/2_) represent the nZVI on the MAC. Moreover, the peaks (Figure 2d) appear at 56 eV for Fe 3p and at 103 eV for Fe 3s, indicating the presence of the FexOy or FexCy phases in the nZVI–MAC, respectively. This result corresponds to the above-mentioned characterization of the carbon oxide thin layer structure and iron carbide in the material.

Additionally, at Raman analysis (see ESI, Figure S3), two characteristic peaks at 1326 cm^−1^ and 1599 cm^−1^ for the activated carbon samples can be observed, corresponding to D and G bands, respectively [35]. The ratio increases from 1.15 (MAC) to 4.07 (nZVI–MAC) after nZVI was loaded, representing the reduction of the ordered carbon structure. Two characteristic peaks of graphite significantly decreased in intensity, which should be because the surface iron further damaged the original ordered sp2-bonded carbon domains of the activated carbon material, greatly increasing the material’s degree of defects and greatly increasing the number of reactive sites.

### 2.2. Performance of nZVI-MAC for Cr(VI) Removal

The effects of different parameters, such as pH, contact time, initial concentration of pollutant and reagents used were studied to evaluate the properties of the materials. The solution pH is one of the most important parameters that influences the surface charge [40]. During this study, from pH 2 to pH 12, the removal rate decreased imperceptibly from 98.50% to 96.21%. Previous studies [16,17,45,46] have reached the same conclusion that an acidic environment is suitable for removing Cr(VI):

(1) $$14H^{+}+{\mathrm{CrO}}_{7}^{2-}+3{\mathrm{Fe}}^{0}\to 3{\mathrm{Fe}}^{2+}+2{\mathrm{Cr}}^{3+}+7H_{2}O$$

(2) $$14H^{+}+{\mathrm{CrO}}_{7}^{2-}+6{\mathrm{Fe}}^{2+}\to 6{\mathrm{Fe}}^{3+}+2{\mathrm{Cr}}^{3+}+7H_{2}O$$

(3) $$\left(1-x\right){\mathrm{Fe}}^{3+}+{\mathrm{xCr}}^{3+}+3H_{2}O\to {\mathrm{Cr}}_{x}{\mathrm{Fe}}_{1-x}{\left(\mathrm{OH}\right)}_{3}+3H^{+}$$

(4) $$\left(1-x\right){\mathrm{Fe}}^{3+}+{\mathrm{xCr}}^{3+}+2H_{2}O\to {\mathrm{Cr}}_{x}{\mathrm{Fe}}_{1-x}\mathrm{OOH}+3H^{+}$$

Considering the above reactions in the system, an increase in the H^+^ concentration can cause Reactions (1) and (2) to move in the positive reaction direction, thereby causing a large amount of Cr(VI) to be converted to Cr(III). Thus, a great amount of Cr(III) is coprecipitated with trivalent iron ions to produce insoluble iron-chromium hydroxides (such as ${\mathrm{Cr}}_{x}{\mathrm{Fe}}_{1-x}{\left(\mathrm{OH}\right)}_{3}$ and ${\mathrm{Cr}}_{x}{\mathrm{Fe}}_{1-x}\mathrm{OOH}$). During previous studies, ZVI particles have significantly affected the removal of Cr(VI) or other contaminants only when the system is at pH ≤ 6 [16,17,30,47]. However, in this study, the Cr(VI) removal rate of the material was reduced as the pH increased, but the reduction rate was very small. This finding may be because more nZVI particles loaded on the material compensates for the lack of hydrogen ions at a higher pH.

**Figure 3:**
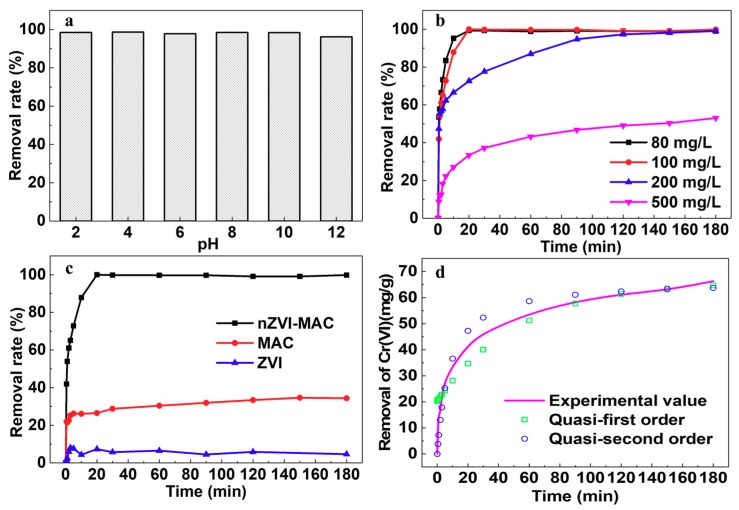
The pH (**a**), initial concentration (**b**), the different reagents of nZVI-MAC/MAC/nZVI (**c**) effect on the removal of Cr(VI) by nZVI-MAC (**d**), adsorption kinetics of Cr(VI) by nZVI-MAC (**a**: initial concentration: 100 mg/L; reagent dosage: 0.2 g/50 mL; reaction equilibrium time: 3 h; temperature: 25 °C; **b**: pH: 4; reagent dosage: 2 g/500 mL; oscillation speed: 130 rpm; temperature: 25 °C; **c**: initial concentration: 100 mg/L; pH: 4; reagent dosage: 2 g/500 mL; oscillation speed: 130 rpm; temperature: 25 °C; **d**: initial concentration: 500 mg/L; pH: 4; reagent dosage: 2 g/500 mL; oscillation speed: 130 rpm; temperature: 25 °C).

The initial experimental conditions are as above, with initial Cr(VI) concentrations of 80–500 mg/L. The results (Figure 3b) showed that the removal rate decreased from 99.84% to 53.06%, with an increase in the initial Cr(VI) concentration, and saturation removal was 66 mg/g. Compared with reports in the past three years, this study found that the treatment capacity of this material for chromium was better than that of most studies, only worse than that of two biochar supported nZVI [33,48] and one chitosan supported nZVI [49] (see ESI, Table S2). Subsequently, the removal rates of Cr(VI) at different times for different materials (nZVI–MAC, MAC, ZVI) also were investigated. Figure 3c shows the comparison of MAC and ZVI on Cr(VI) removal, and the optimal reaction system is found to be nZVI–MAC. MAC or ZVI alone were not able to achieve the effect of their combination in the removal of Cr(VI), and the removal of Cr(VI) from the nZVI–MAC material does not appear to be a simple summation of the two material’s effects. According to previous studies, the removal of Cr(VI) by ZVI mainly includes two processes, namely, the reduction process of Cr(VI) by ZVI and the co-precipitation process of Cr(III) and Fe^3+^ [17]. The removal of Cr(VI) from activated carbon is mainly due to the high adsorption of the material. Studies [17,50,51] have shown the formation of a micro-electrolysis effect between iron and carbon, hence accelerating the reaction and enhancing the removal efficiency.

Through adsorption kinetics analysis (Figure 3d), we found that the adsorption process of Cr(VI) removal by nZVI-MAC was more consistent with the quasi-second order adsorption kinetics equation (R^2^ = 0.9955), which was a chemical adsorption process. The redox reaction between nZVI and Cr(VI) resulted in the formation of insoluble Cr(III), and the iron ions formed ferric chromium hydroxide insoluble matter with Cr(III) in the solution, which accelerated the deposition of pollutants. However, the reaction not only contains the above reduction and co-precipitation processes, but also complex diffusional and chemical mass-transfer phenomena. Giorgio et al. studied the role of peroxide in Fe(0) consumption, and established the classical “shrinking core model” equation, which well describes the reduction process of nZVI particles, considering the diffusion and chemical mass-transfer process [52]. This provides a good idea for describing the whole process of ZVI reduction in the future. The removal of Cr(VI) by magnetic iron materials is similar to that of nZVI [8,53,54]. The removal of Cr(VI) by magnetic carbon materials is also a mixed adsorption-reduction process: the adsorption of carbon materials is the main effect, while the presence of Fe_3_O_4_ has a synergistic effect on Cr(VI) removal. Different from the Cr removal process of nZVI, the composite materials mentioned above are more suitable for material reuse due to their main role in adsorption, which greatly reduces the cost of the material itself, but the cost of the material recovery process has to be considered.

### 2.3. Mechanisms of Cr(VI) Removal

**Figure 4:**
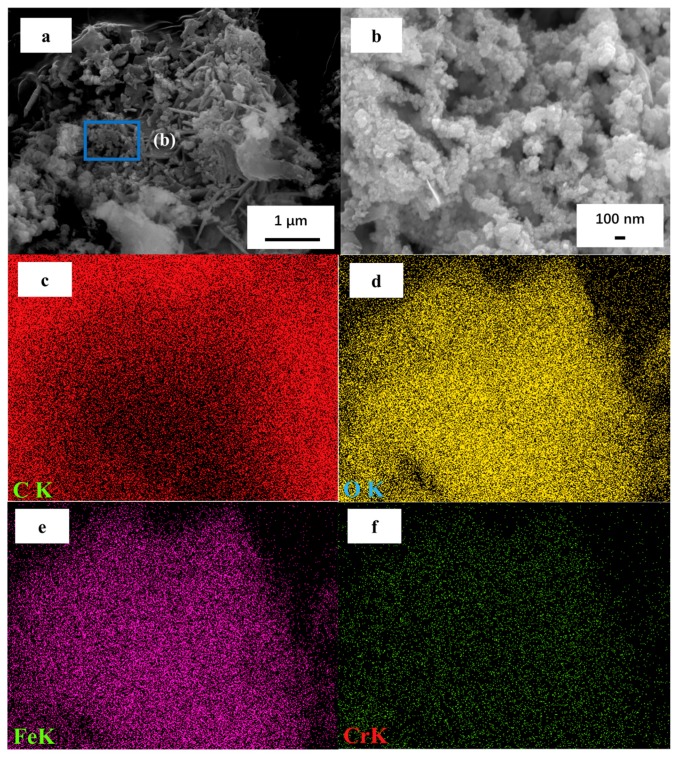
nZVI–MAC after Cr(VI) treatment: (**a**,**b**) SEM images (**b** is the amplification image of the corresponding dotted box in **a**); (**c**,**f**) EDS mapping of (**a**).

To investigate the typical removal mechanisms of Cr(VI) ions by nZVI–MAC, the morphologies and chemical compositions before and after Cr(VI) ion removal were compared. Viewing the scanning electron microscope (SEM) characterization (Figure 4a), it shows that a microstructure almost remains in the original morphology, indicating a good structure and stability. The enlarged SEM image (Figure 4b) clearly shows that a large number of nZVI chains twining on the surface of MAC were maintained. However, the surface of the chains is covered by thick precipitates, and the boundaries between chains are blurred. Some parts of the surface display a large group of flocculent precipitation. Considering previous studies [45,46] and the above prediction, the precipitate should be iron-chromium hydroxide. Additionally, EDS mapping analyses (Figure 4c–f) of nZVI–MAC after Cr(VI) treatment also showed the appearance of C, O, Fe and Cr signals. Furthermore, the features corresponding to the last three elements (O, Fe and Cr) are highly consistent in position, indicating that Cr(VI) forms iron-chromium hydroxides during the reduction of nZVI. However, the position of the C feature is precisely the opposite of the above. The above structural analysis shows that chromium and iron redox products are coprecipitated on the surface of the material and that the surface is tightly coated with this coprecipitate. The C content of the material does not participate during the reaction but functions only as an adsorption component and the inert electrode component in the micro-electrolysis process [17].

**Figure 5:**
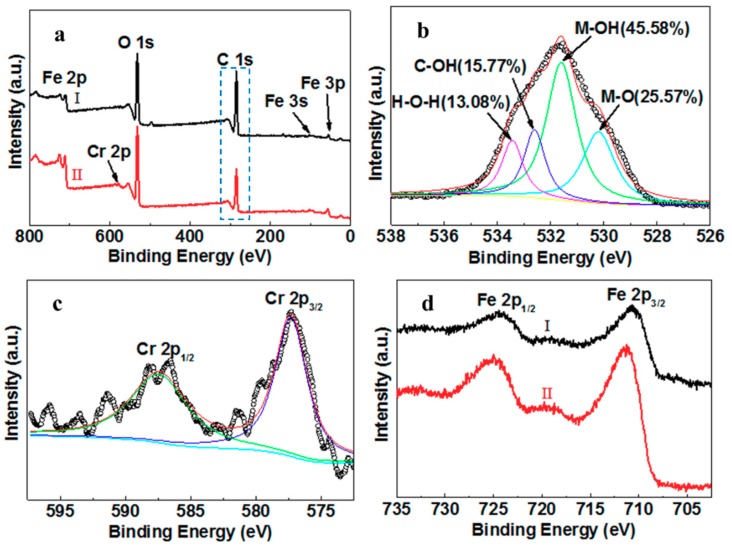
(**a**) XPS spectra of nZVI–MAC before (I) and after (II) Cr(VI) treatment; (**b**) C 1s spectrum after Cr(VI) treatment; (**c**) Cr 2p spectrum; (**d**) Fe 2p before (I) and after Cr(VI) treatment (II).

To better understand the mechanisms of Cr(VI) removal, the elemental composition of the nZVI–MAC composites before (I) and after (II) Cr(VI) adsorption was further analysed by XPS (Figure 5a). The C 1s peak of nZVI–MAC greatly decreased, while a new peak assigned to Cr 2p appeared due to the fast reduction of Cr(VI) by ZVI, and the produced Cr(III) was coprecipitated with iron ions on the surface. The spectrum of Cr 2p (Figure 5c) can be fitted by two peaks at 587.5 for Cr 2p_1/2_ and 577.5 eV for Cr 2p_3/2_, indicating that Cr(VI) is reduced to Cr(III). After the removal of Cr(VI) ions, the ratio of M–OH increased from 21.38% to 45.58%, and C–OH decreased from 35.53% to 15.77%, indicating that –OH and –COOH may be involved in the removal of Cr(VI).

Additionally, the ratio of M–O increased from 7.86% to 25.57% after the removal of Cr(VI) ions, which might be due to the formation of some new metal oxides after Cr(VI) removal, such as Cr–O [35]. The ratio of the peak at 533.45 eV changed more substantially, suggesting that adsorbed H_2_O was involved in the removal process, which may be caused by a large amount of adsorbed water that is consumed by the coprecipitation process (Figure 5b), according to Reactions (3) and (4). After the removal of Cr(VI), the Fe 2p_1/2_ and Fe 2p_3/2_ peaks changed to higher binding energies (Figure 5d), which represents a possible combination of –OH and Fe in the removal process and that iron hydroxides and iron oxides [16,19,35], such as ${\mathrm{Cr}}_{x}{\mathrm{Fe}}_{1-x}\mathrm{OOH}$, ${\mathrm{Cr}}_{x}{\mathrm{Fe}}_{1-x}{\left(\mathrm{OH}\right)}_{3}$, FeOOH, and ${\mathrm{Fe}}_{2}O_{3}$, are formed in the reaction.

The XRD patterns before and after the Cr(VI) removal process (Figure S4) clearly show that new diffraction peaks appeared and that some diffraction peaks disappeared. The characteristic peaks (003, 101, 012, 018) of potassium chromium oxide (PDF#28-0745) appeared in the XRD spectrum of nZVI–MAC after the Cr(VI) removal process, corresponding to the generation of Cr(III). However, a peak of chromium oxide (011) (PDF#65-1388) appears in Figure S4b, indicating the presence of Cr(VI) that has not been reduced but has been adsorbed by the material. Simultaneously, the material surface showed a large number of diffraction peaks for iron-chromium compounds (Figure S4b), such as iron chromium oxide hydrate (126) (PDF#76-0697), iron chromium oxide (220) (PDF#24-0511), and chromium iron oxide (104, 024) (PDF#35-1112), demonstrating that Cr(III) and iron ions produced a large amount of coprecipitates during the removal process that covered the material surface. Additionally, iron that did not react with Cr(VI) at the material surface was oxidized in the reaction process and produced hydroxyl iron oxides and iron oxides, such as FeOOH (021, 221, 152) (PDF#70-0714) and Fe_2_O_3_ (4,0,12) (PDF#25-1402). Furthermore, compared with nZVI–MAC before the reaction, the crystal structure of nZVI–MAC after the reaction underwent many changes. The characteristic peaks of ZVI were weakened significantly, such as the (110) diffraction peaks, and the (200, 211) diffraction peaks nearly disappeared, indicating that ZVI almost completely reacted in the reaction process. The fullerite-like carbon component (210, 113) completely disappeared after the reaction, possibly because the carbon component is covered by the coprecipitated substances.

Finally, the Fourier transform infrared spectra(FT-IR) of nZVI–MAC before and after chromium (VI) treatment were compared in the supporting information (ESI, Figure S5). The infrared peak at 3434 cm^−1^ before the treatment, which is assigned to –OH stretching vibrations, shifts to 3348 cm^−1^ after the treatment, the C–OH stretching vibration (1379 cm^−1^) widens after the treatment, and the C–O stretching vibrations (1030 cm^−1^) shifts to 1021 cm^−1^ after the treatment. These changes in the location and morphology of infrared peaks may be due to the formation of hydrogen bonds between ferric chromium hydroxide and the “free”–OH group, as well as skeletal vibration of the bonded –OH bonds of carbon [35]. The activated carbon skeleton vibration (1628 cm^−1^) was the same before and after the reaction, indicating that the material is thermally stable. Moreover, the C–H (2900 cm^−1^) stretching vibration peaks on the carbon skeleton almost disappeared after the reaction, possibly because the resulting ferrochrome hydroxide coprecipitate covered the surface of the material.

## 3. Materials and Methods

### 3.1. Materials and Instruments

Activated carbon powder (100 mesh, 99.9%) was supplied by Aladdin Biochemical Technology Co., Ltd., Shanghai, China. Microcrystalline cellulose (MC) (50 μm, 99.99%) was purchased from Acros Organics Co., Ltd., Morris Plains, NJ, USA. Polyethylene glycol (HO(CH_2_CH_2_O)_n_H, 3600–4400) was supplied by Guangfu Fine Chemical Research Institute, Tianjin, China. Ferrous sulfate (FeSO_4_·H_2_O, ≥99%) was purchased from Fuchen Chemical Reagents Factory, Tianjin, China. Sodium borohydride (NaBH4, ≥98%) was purchased from Huadong Reagents Factory, Tianjin, China. Citric acid (C_6_H_8_O_7_, ≥99.5%), ethanol (C_2_H_6_O, ≥99.7%), sodium hydroxide (NaOH, ≥96%) and sulfuric acid (H_2_SO_4_, 95–98%) were obtained from Chemical Works Co., Ltd., Beijing, China. All materials were of analytical grade. Ultrapure water was used throughout the experiments.

SEM and EDS (JSM-7001F, JEOL, Tokyo, Japan), HRTEM (JEM-2010, JEOL, Tokyo, Japan), XRD (Ultima IV, Rigaku, Tokyo, Japan), XPS (ESCALAB 250, Thermo Fisher Scientific, Waltham, MA, USA), a Raman spectrometer (inVia, Renishaw, Wotton-under-Edge, UK), FT-IR (Tensor 27, Bruker, Karlsruhe, Germany), Specific surface area analyzer (ASAP 2020, Micromeritics, Norcross, GA, USA), Laser particle size analyzer (Mastersizer 3000, Malvern, Malvern, UK), a Ultraviolet and visible(UV-Vis) spectrophotometer (TU-1900, General instrument, Beijing, China), an incubator shaker (HZQ-F160, Yiheng scientific instrument, Shanghai, China), and a vacuum freeze dryer (SCIENTZ-18N, New zhi biotechnology, Ningbo, China).

### 3.2. Preparation of MAC

Preparation of MAC was carried out in the following steps. Activated carbon powder (500 mg) and citric acid (220 mg) were added to 30 mL of an MC suspension (12 g/L), which was then shaken with ultrasonic vibration for 30 min and stirred in an incubator shaker for 4 h. The suspension was subsequently transferred into a 50 mL poly (para-phenol)-lined autoclave and heated at 220 °C for 12 h to obtain MAC. Finally, the product was washed by ethanol, centrifuged and dried before it was used.

### 3.3. Preparation of nZVI–MAC in a Polyethylene Glycol–H_2_O Solution

The nZVI–MAC composites were prepared by a conventional reduction reaction, but the synthetic solution was changed from ethanol–H_2_O to polyethylene glycol–H_2_O. First, a ferrous solution was prepared by 7.45 g of FeSO_4_·7H_2_O into 100 mL of a polyethylene glycol–H_2_O solution (15 g/L) in a dry spherical flask and shaking it with ultrasonic vibration for 30 min. Subsequently, the prepared MAC powder (1 g) was added to the ferrous solution and stirred magnetically for 30 min. A NaBH_4_ solution (4.05 g of NaBH_4_ dissolved in 100 mL of water) was then added for approximately 40 min, and the sample was continuously stirred for approximately 30 min under a nitrogen atmosphere. Finally, the suspension was separated using a centrifuge, washed with pure ethanol and water more than three times, and dried for 10 h in a vacuum freeze dryer.

### 3.4. Characterization

SEM and EDS analyses were performed with a field emission scanning electron microscope at an accelerating voltage of 15 kV. HRTEM images were obtained with a microscope operating at an accelerating voltage of 200 kV. The phase and crystal structure were analysed by XRD with Cu–Ka radiation. XPS analyses were conducted on an X-ray photoelectron spectrometer equipped with a focused monochromatic Al Ka X-ray source (150 W), and calibrated with reference to the C 1s peak (284.8 eV). Raman spectra were obtained on a Micro-Raman Spectrometer at a 633-nm laser excitation. FT-IR spectra of the samples were recorded on an FT-IR spectrophotometer over a wavenumber range of 500–4000 cm^−1^ at a resolution of 4 cm^−1^ using the KBr pellet method. Specific surface area analyzer was used to measure the specific surface area and pore volume. The particle size distribution of materials was measured by a laser particle size analyzer using dry method.

### 3.5. Batch Experiments

Each beaker contained 2 g of reagents and 500 mL of an aqueous Cr(VI) solution (concentrations ranging from 80 to 500 ppm) with various pH values that had been reached by adjustment with a H_2_SO_4_ (1 M) or NaOH (1 M) solution. The final supernatant was filtered through a 0.45 μm filter membrane, and the heavy metal concentrations in the residual were determined by the colorimetric method. The relationship between the standard Cr(VI) solution concentration and the absorbance was established to calculate the adsorption.

The concentration of Cr(VI) in the solution was measured by diphenyl–carbazide spectrophotometry. After the reaction, 0.25 mL of supernatant was filtered into 25 mL colorimetric tubes with 2.0 mL of 20 g/L diphenyl–carbazide as the chromogenic reagent; the solutions were mixed well and allowed to stand for 5 min. Then, 0.5 mL of a H_2_SO_4_ solution (H_2_SO_4_ to water ratio (*v/v*) of 1:1) was added, and the resulting solution was mixed well. Finally, the colorimetric tube was filled to 25 mL with ultrapure water, and measured by a UV–Vis spectrophotometer at 540 nm within 20 min. The residual Cr(VI) concentration and removal rate were calculated by the established Cr(VI) standard curves. The effects of solution pH values, Cr(VI) concentrations, times and load ratios of nZVI–MAC were studied.

## 4. Conclusions

To summarize, nZVI–MAC composites were successfully synthesized by completely replacing the traditional ethanol solution with a glycol solution in the synthesis process, in which nZVI was loaded on the surface of MAC materials with a bead-string structure. Polyethylene glycol played a key role in the loading process of nZVI, providing a large number of hydroxyl functional groups rather than ethanol to ensure the stable dispersion of nanoparticles on the MAC surface. The uniquely structured composites exhibited an outstanding remediation performance towards Cr(VI) ions in contaminated water. The treatment rate of Cr(VI) with low concentration (80–100 mg/L) was over 99%, the maximum saturation removal was up to 66 mg/g, and the material was applicable over an extensive pH range (from 2 to 12, the treatment effect was over 96%), which guarantees their further application prospects in wastewater remediation. The removal processing of Cr(VI) by nZVI–MAC involves the integration of a redox reaction and a coprecipitation reaction and conforms to the quasi-second order adsorption kinetics equation (mainly chemical adsorption process), which is attributed to the multi-active sites of the MAC materials and the special combination of iron and carbon, which constitute a micro-electrolysis process in the system. Cr(VI) forms iron–chromium hydroxides during the reduction of nZVI, and these redox products are coprecipitated on the surface of the material.

## Supplementary Materials

The following are available online at https://www.mdpi.com/1420-3049/25/1/47/s1, Figures S1–S6, Tables S1 and S2 [11,26,33,48,49,55,56,57,58,59,60,61,62].
