# Supplementary material for: Preparation of Activated Carbon Supported Bead String Structure Nano Zero Valent Iron in a Polyethylene Glycol-Aqueous Solution and Its Efficient Treatment of Cr(VI) Wastewater
Source: Molecules. 2019 Dec 21;25(1):47. doi: 10.3390/molecules25010047 (PMC6982729; doi:10.3390/molecules25010047)
Supplement: Supplementary file 1 [file molecules-25-00047-s001.pdf]

molecules

Supplementary Material

Preparation of Activated Carbon Supported Bead String Structure Nano Zero Valent Iron in a Polyethylene Glycol-Aqueous Solution and Its Efficient Treatment of Cr(VI) Wastewater

Chunlei Jiao [1,2], Xiao Tan [1], Aijun Lin [1] and Wenjie Yang [3,4,*]

1 Department of Environmental Science and Engineering, Beijing University of Chemical Technology, Beijing 100029, China; jiaocl@ihep.ac.cn (C.J.); tanxiaobuct@163.com (X.T.); linaj@mail.buct.edu.cn (A.L)
2 Institute of High Energy Physics, Chinese Academy of Sciences, Beijing 100049, China
3 College of Renewable Energy, North China Electric Power University, Beijing 102206, China
4 Chinese Academy for Environmental Planning, Beijing 100012, China
* Correspondence: yangwj@caep.org.cn (W.Y.)

Academic Editor: Giorgio Vilardi
Received: 15 November 2019; Accepted: 18 December 2019; Published: date

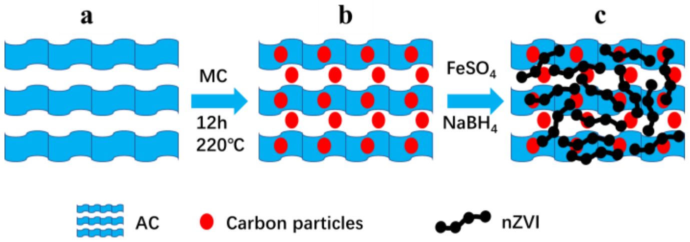

Figure S1. Schematic illustration of the preparation process of nZVI-MAC: (**a**) activated carbon (AC); (**b**) MAC in the presence of microcrystalline cellulose; and (**c**) in situ growth of nZVI on the MAC skeleton.

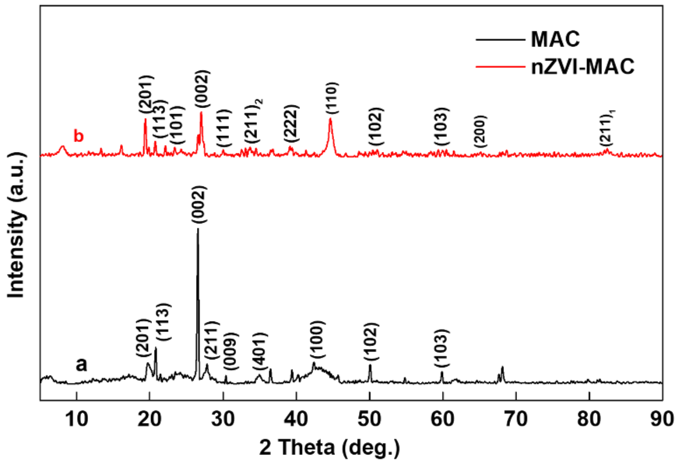

Figure S2. XRD patterns of the as-prepared samples: (**a**) MAC and (**b**) nZVI-MAC.

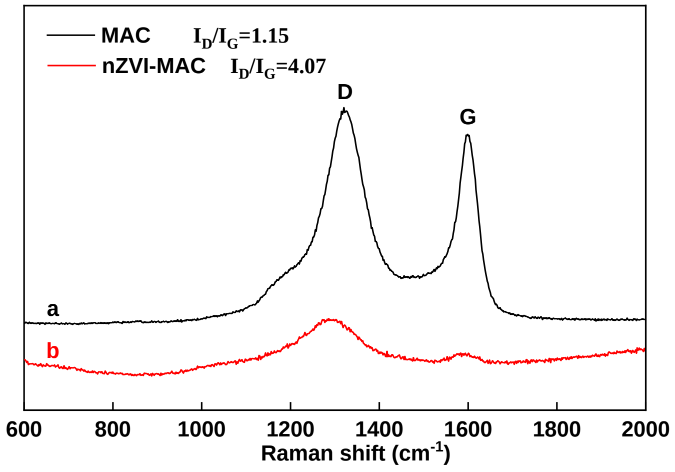

Figure S3. Raman spectra of the as-prepared samples: (**a**) MAC and (**b**) nZVI-MAC.

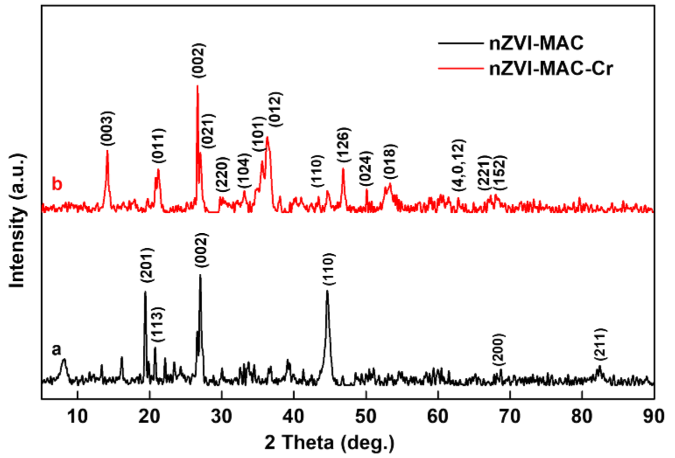

Figure S4. XRD patterns of nZVI-MAC samples before (**a**) and after Cr(VI) treatment (**b**).

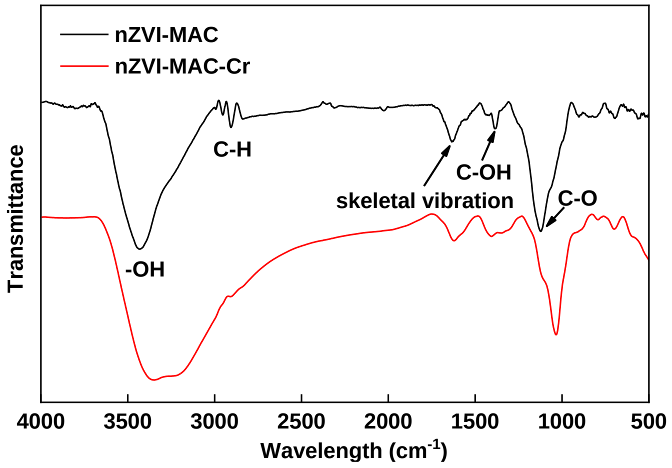

Figure S5. FT-IR spectra of nZVI–MAC samples before and after Cr(VI) treatment.

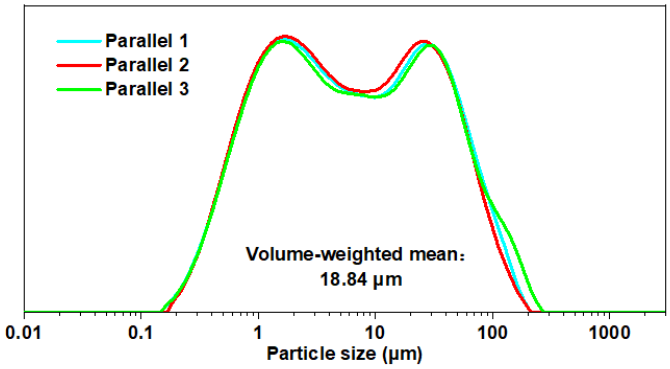

Figure S6. Particle size distribution of nZVI–MAC composite materials.

Table S1. Specific surface area and pore volume of material.

	Traditional activated carbon	MAC	nZVI-MAC
Specific surface area (m^2/g)	57.30	59.93	141.85
Pore volume (cm^3/g)	0.227	0.207	0.202

Table 2S. Comparison of the performance of carbon materials supported nZVI for chromium removal in previous studies of past three years.

**Material type**	**Synthetic method**	**Treatment capacity**	**Reference**
magnetic Fe/C crosslinked nanoparticles	chemical reduction co-precipitation and the residue hot air in-situ oxidation method	48.0 mg/g	[55]
Activated carbon impregnated by nZVI	incipient wetness method	5.07 mg/g	[56]
Sewage sludge-derived biochar immobilized nZVI	liquid-phase reduction	18.0 mg/g	[57]
Reduced graphene oxide- nZVI	liquid-phase reduction	50.0 mg/g	[58]
Mixed-iron coated olive stone bio-sorbent particles	liquid-phase reduction	12.66 mg/g	[26]
nZVI supported by biochar	liquid-phase reduction	125.22 mg/g	[33]
nZVI-carbon nanotubes	liquid-phase reduction	2.70 mg/g	[59]
nZVI nanoparticles embedded into reduced graphene oxide-alginate bead	liquid-phase reduction	0.67 mg/g	[60]
Activated carbon fiber supported nZVI	liquid-phase reduction	16.03 mg/g	[11]
Sewage sludge based activated carbons impregnated with nZVI	liquid-phase reduction	42.0 mg/g	[61]
nZVI supported on herb-residue biochar	liquid-phase reduction	107.4 mg/g	[48]
nZVI supported by rice straw biochars	liquid-phase reduction	40.0 mg/g	[62]
Chitosan nZVI nanoparticles	liquid-phase reduction	142.8 mg/g	[49]
Activated carbon supported bead string structure nZVI	liquid-phase reduction	66.0 mg/g	this study
